# Three-dimensional models of skeletal muscle under tension: a systematic review and core outcome set

**DOI:** 10.1038/s41536-026-00464-z

**Published:** 2026-04-14

**Authors:** Kunal Bhanot, Core Outcome Set Review Group, George Truskey, Nenad Bursac, Antonio Musarò, Roger Kamm, Ng Shyh-Chang, Lieven Thorrez, Kazunori Shimizu, Herman Vandenburgh, Neal Thurley, Robin O. Cleveland, Jolet Mimpen, Sarah J. B. Snelling, Rory Rickard, Robert Staruch

**Affiliations:** 1 Academic Department of Military Surgery and Trauma, Birmingham, UK; 2 Department of Plastic Surgery, John Radcliffe Hospital, Headley Way, Oxford, UK; 3 Bodleian Healthcare Libraries, University of Oxford, Oxford, UK; 4 Institute of Biomedical Engineering, University of Oxford, Oxford, UK; 5 Botnar Research Institute, Nuffield Department of Orthopaedics, Rheumatology and Musculoskeletal Sciences, Oxford, UK; 6 Department of Biomedical Engineering, Duke University, Durham, NC, USA; 7 DAHFMO Unit of Histology and Medical Embryology, University of Rome, Rome, Italy; 8 Departments of Mechanical Engineering and Biological Engineering, Massachusetts Institute of Technology, Cambridge, MA, USA; 9 Laboratory of Organ Regeneration and Reconstruction, Institute of Zoology, Chinese Academy of Sciences, Beijing, China; 10 Department of Development and Regeneration, KU Leuven, Leuven, Belgium; 11 Department of Biomolecular Engineering, Nagoya University, Nagoya, Japan; 12 Department of Pathology and Laboratory Medicine, Brown University, Providence, RI, USA

**Keywords:** Tissue engineering, Tissues

## Abstract

Biologically representative three-dimensional (3D) skeletal muscle models are required to understand the mechanisms underpinning muscle disease. The primary aim of this systematic review is to evaluate the current literature on 3D skeletal muscle models under tension. Its secondary aim is to explore injury mechanisms and generate a novel Core Outcome Set (COS) for reporting of such models. In vitro studies that utilised any skeletal muscle cell types cultured with an anchor system imposing axial strain were eligible for inclusion. A modified Delphi approach using corresponding authors of included studies was then developed to obtain a COS. This systematic review was reported in compliance with the PRISMA 2020 checklist. 37 articles were included. Of these, 7 articles induced an injury in their model. Cell lines used were both human and animal. Immunohistochemical testing on models revealed greatest concordance for myosin heavy chain (MHC), alpha actinin, and Pax7 as a means of demonstrating striated muscle. The final COS agreed on multiple reporting criteria for the validation of models based on morphology, phenotype and function. This systematic review summarises the current literature and has developed core outcome reporting for models of 3D skeletal muscle that impose axial strain.

## Introduction

Skeletal muscle is a highly organised and multifunctional tissue composed of myofibrils that generate movement through coordinated contractile force. Its architecture integrates vascular, neural, and stromal networks within a specialised extracellular matrix (ECM) to enable precise motor control and repair^1^. In vitro systems that recapitulate this complexity are essential for advancing our understanding of muscle disease, regeneration, and tissue engineering. Although in vivo models have been instrumental in elucidating pathophysiological mechanisms, they are costly, time-intensive, and offer limited throughput for early-stage discovery. In contrast, human cell–based in vitro models provide a more accessible and ethically sustainable platform for probing human-specific biology.

A defining feature of a physiologically relevant in vitro muscle model is its capacity for self-regeneration following injury—a hallmark of structural and functional maturity. The ability to undergo autonomous repair implies the presence of key cellular constituents, including satellite cells and their regulatory niche. Incorporating controlled injury paradigms into three-dimensional (3D) muscle constructs, therefore, represents a critical step toward developing models that emulate the native regenerative cycle. Unlike animal recovery models, which are constrained by ethical considerations and species-specific variability, in vitro platforms enable precise manipulation of injury type, duration, and severity, and can be maintained longitudinally to study repair dynamics.

3D culture systems are increasingly being adopted for drug discovery, disease modelling, and mechanobiological research^2^. However, their clinical translatability remains limited by inconsistent validation methods and heterogeneous reporting standards^3^. Current approaches to skeletal muscle modelling—including explanted tissue, microfluidic systems, and hydrogel-based constructs—have demonstrated the capacity to reproduce structural organisation and contractile function. The application of mechanical tension within these systems is particularly important: it promotes myofibre alignment, enhances sarcomeric gene expression, and drives the emergence of functional contractile units^4^. Indeed, tensioned constructs display greater force production, more mature morphology, and improved mitochondrial and metabolic function compared with 2D cultures^5^. These findings underscore that mechanical load is not merely structural but an essential driver of myogenesis.

Despite rapid innovation, the absence of methodological standardisation has hindered cross-comparison between studies and slowed clinical translation. To date, no systematic synthesis of 3D skeletal muscle models under tension has been published, nor has a consensus emerged regarding how to reproducibly induce and assess injury within these systems. This review, therefore, aims to evaluate the current landscape of 3D muscle models under tension, examine the experimental strategies used to simulate injury and identify a core set of outcome measures that can guide future model validation and reporting.

The concept of Core Outcome Sets (COS) originates from clinical research, where they are used to establish uniform outcome measures that facilitate comparability across trials^6,7^. COS frameworks reduce reporting heterogeneity, minimise outcome bias, and improve the synthesis of evidence. These same principles are increasingly relevant to preclinical and in vitro research, where variability in outcome selection and reporting often precludes meaningful comparison between studies.

Applying COS methodology to in vitro bioengineering could provide a standardised foundation for evaluating model performance and translational potential. In the context of skeletal muscle, developing a reproducible, morphologically and functionally validated model against an agreed set of outcomes would represent a pivotal step forward. Such consensus would not constrain innovation but rather provide a benchmark for reproducibility and comparability—ensuring that novel systems can be reliably assessed within a shared framework.

By defining essential morphological, molecular, and functional outcomes, a COS would enable model standardisation during both development and application phases. This, in turn, would accelerate translation from experimental platforms to clinically relevant systems. Although COS frameworks have been widely implemented in clinical research, they have yet to be proposed for in vitro modelling. The present review, therefore, introduces, for the first time, a COS tailored to 3D skeletal muscle models under tension—establishing a foundational step toward harmonised, reproducible, and translatable in vitro muscle science.

## Results

**Fig. 1:**
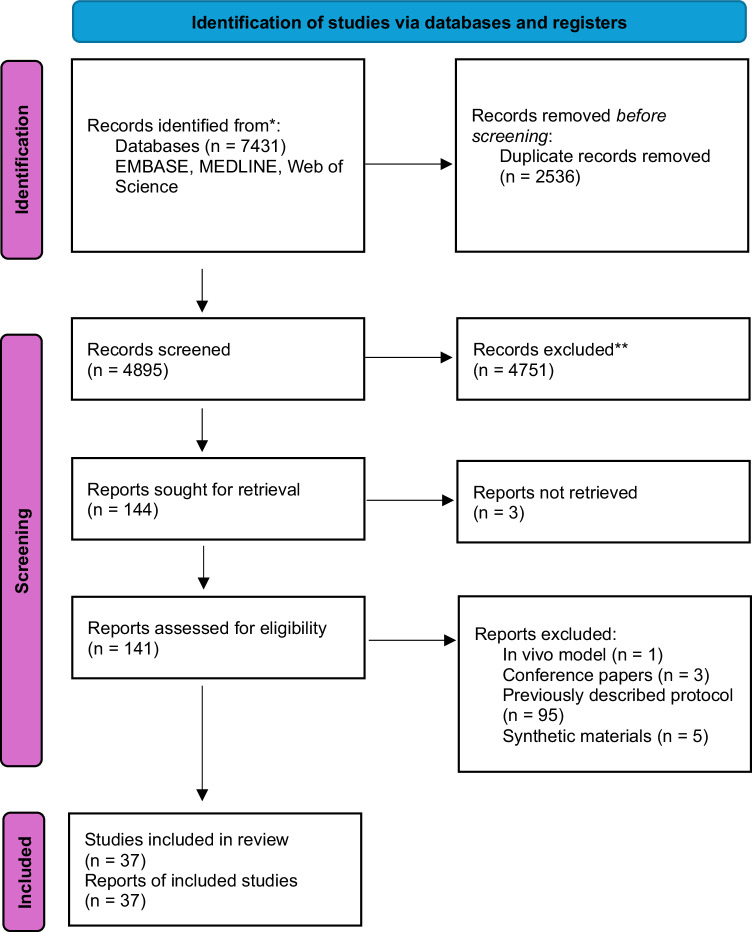
PRISMA flowchart detailing screening process and final number of included studies. *Consider, if feasible to do so, reporting the number of records identified from each database or register searched. **No automation tools were used.

The MEDLINE search retrieved 2650 records. 1970 records were retrieved from EMBASE. Web of Science produced 2811 records. The total number of articles found from the above search strategies was 4895 (Fig. 1). Through abstract review and bibliographic screening, a total of 141 articles were included for final full-text screening. Following this, 37 articles that described models of 3D skeletal muscle using anchors were included in this systematic review. Of these, 7 articles induced an injury in their model.

### Study characteristics

All articles were experimental reports of in vitro models. Studies were published between 1996 and 2024.

### Cell types

**Fig. 2:**
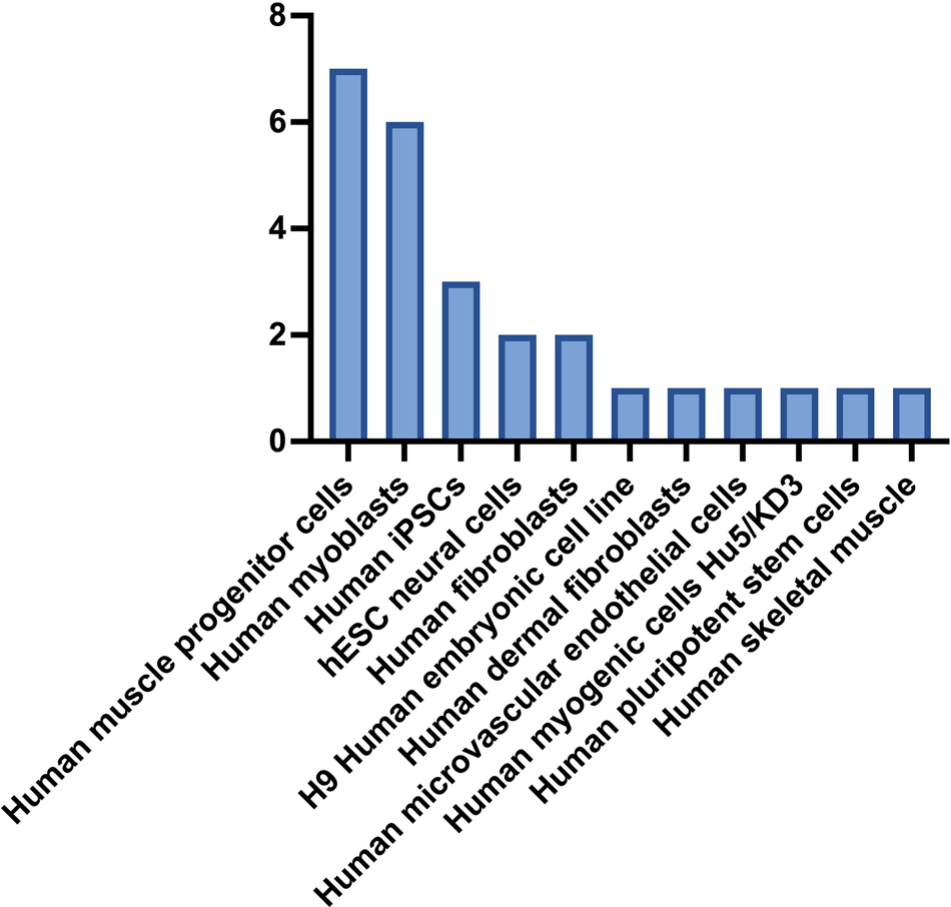
Human cell lines incorporated within vitro 3D muscle model. Bar depicts number of models using specified human cell line.

**Fig. 3:**
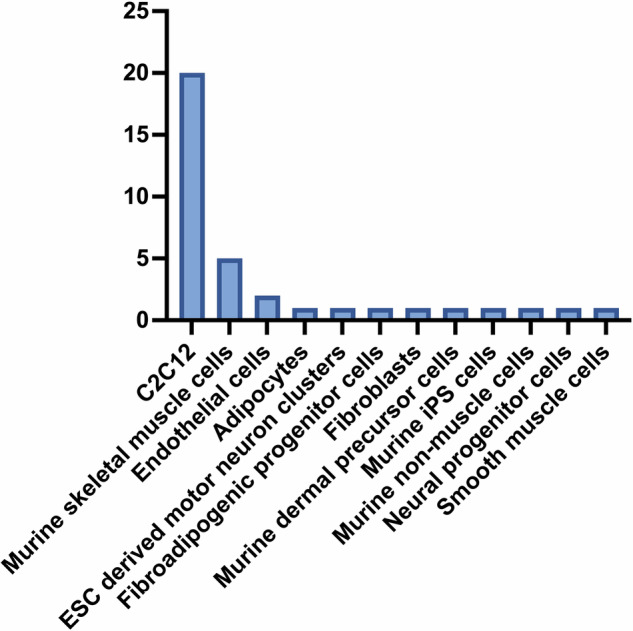
Animal cell lines incorporated within a 3D in vitro muscle model. Bar depicts number of models using specified animal cell line.

**Fig. 4:**
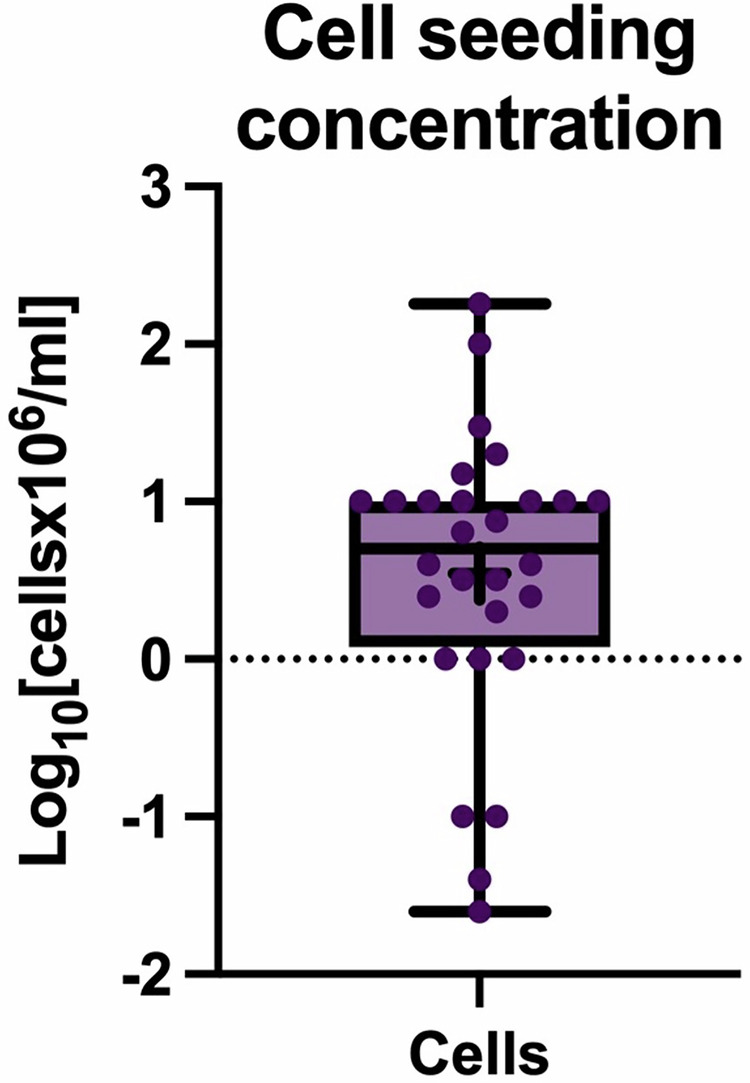
Cell seeding concentrations for 3D in vitro muscle models with box depicting interquartile range and cross indicating mean. Individual values as dots.

The 37 articles utilised 12 different animal cell types, 11 different human cell types or a combination of both (Figs. 2 and 3). Whilst the majority of cells were of muscle, non-muscle cells such as adipocytes and fibroblasts were also used in conjunction with muscle cells in some models. The murine continuous C2C12 cell was the most common type of animal muscle cell adopted across all studies (*n* = 20) and human muscle progenitor cells were the most common human cells utilised (*n* = 7). The majority of models (*n* = 32) were multi-cellular models. Cell seeding concentration varied considerably across studies (mean 1.62 × 10^7^ cells/ml, range 2.5 × 10^4^ cells/ml to 1.8 × 10^8^ cells/ml (Fig. 4).

### Hydrogel manufacture

**Fig. 5:**
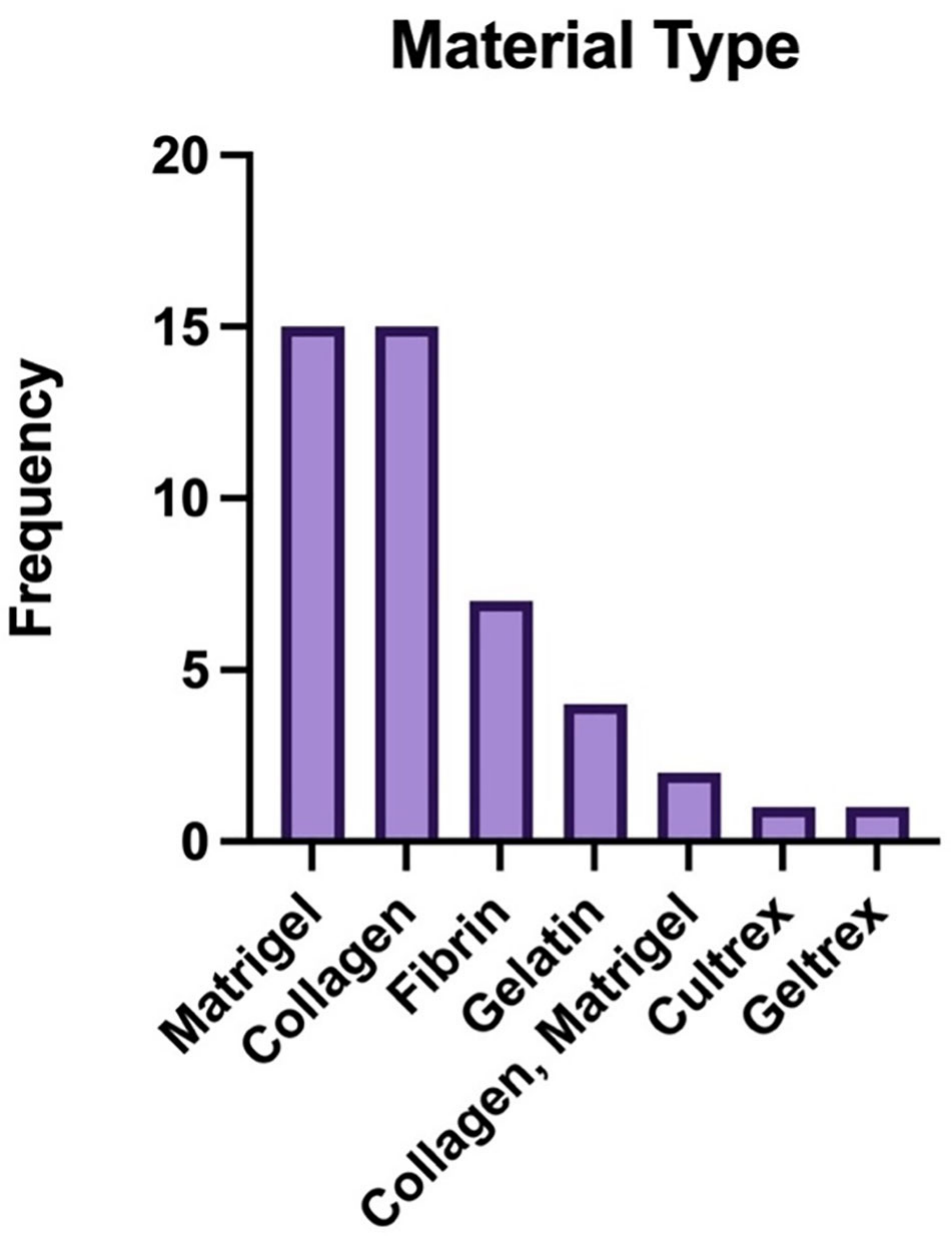
Types of material used for hydrogel manufacture. Bar depicts number of models using specified material.

All studies utilised hydrogels capable of biodegradation. Hydrogels were manufactured from fibrin, collagen, gelatin or other collagen-based basement membrane extracts such as Matrigel™. Collagen and Matrigel™ were the most common types of material used for hydrogel manufacture (Fig. 5).

### Anchor type

18 different techniques were used to anchor hydrogels in cell culture, predominantly using polydimethylsiloxane (PDMS) casted posts (Table 1). There were 6 different iterations of PDMS casts used, with a mould being the most common (*n* = 9). Stainless steel pins were the most common non-PDMS type of anchor used (*n* = 8). Other types of non-PDMS anchor included nylon hooks and fibrin anchors.

**Table 1:** 3D in vitro models under tension

Paper demographics		Model				qPCR and Gene Expression	Histology and IHC	Force measurement
Authors	Year	3D Material	Posts	Cell Type(s)	Cell seeding concentration			
Vandenburgh et al.^24^	1996	Collagen	Velcro or stainless steel pins	C2C12	1–4 ×10^6^ cells/ml	N/A	Tropomyosin	Muscle displacement by microscopy
Okano et al.^25^	1997	Collagen	Fabrication into rod shaped mould	C2C12	3 ×10^7^ cells/ml	N/A	N/A	Muscle displacement by microscopy
Cheema et al.^26^	2003	Collagen	Polyethylene mesh rotation bars	Smooth muscle cells, human dermal fibroblasts, C2C12	4 ×10^6^ cells/ml	IGF-1, MGF	N/A	Culture force monitor (CFM)
Huang et al.^27^	2004	Fibrin	Stainless steel pins	Primary rat myoblasts	1 ×10^5^ cells/ml	N/A	H&E, MHC, MYOD1	Force transducer
Matsumoto et al.^28^	2007	Fibrin	PLGA sutures	C2C12	2.5 ×10^4^ cells/ml	N/A	Myosin	Muscle displacement by microscopy
Ma et al.^29^	2011	Collagen	Not specified	C2C12	5 ×10^4^ cells/well	N/A	MHC, CD31, vWF, Dystrophin, Neurofilament protein	Muscle displacement by microscopy
Carosio et al.^30^	2013	Collagen	Stainless steel pins	Murine skeletal muscle cells, murine non-muscle cells	Not specified	MyoD, myogenin, pax7, CD31, VE-cadherin, VEGFA	MYOD1, MHC, Laminin, PAX7, Alpha SMA, ERTR7	Force transducer
Juhas et al.^31^	2014	Matrigel	PDMS mould	Murine skeletal muscle cells	1 ×10^7^ cells/ml	N/A	PAX7, MYOD1, Myogenin	Force transducer
Garcia-Parra et al.^32^	2014	Cultrex	Not specified	Murine dermal precursor cells	7.5 ×10^3^ cells/cm^2^	PAX7, MYOD, MYOD1, MyH2, MyH3	MHC	N/A
Snyman et al.^33^	2014	Collagen	Stainless steel pins	C2C12, human myoblasts	6.4 ×10^6^ cells/ml murine, 3.2 ×10^6^ cells/ml human	N/A	Desmin, Phalloidin	N/A
Shimizu et al.^34^	2015	Collagen	PDMS slab	C2C12	1 ×10^7^ cells/ml	N/A	Alpha actinin	Muscle displacement by video microscopy
Madden et al.^35^	2015	Matrigel	PDMS mould	Human muscle precursor cells	15 ×10^6^ cells/ml	N/A	MHC, Desmin, PAX7, Alpha actinin, Laminin, CK, Myogenin	Force transducer
Zhu et al.^36^	2017	Fibrin	Not specified	Murine skeletal muscle cells	2 ×10^3^ cells/population	PAX7, integrin	PAX7	N/A
Thorrez et al.^37^	2018	Fibrin	Stainless steel pins	Primary human skeletal muscle-derived cells	2 ×10^6^ cells/well	N/A	N/A	Force transducer
Osaki et al.^38^	2018	Collagen	Fibrin anchor	C2C12, endothelial cells	1 ×10^7^ cells/ml	ang-1, erb-2	Rhodamine, Phalloidin, Alpha actinin, PECAM-1, Type IV collagen	Muscle displacement by video microscopy
Yeo et al.^39^	2018	Collagen	3-axis robot	C2C12	1 ×10^6^ cells/ml	beta actin, MyoD, Myoh2, myogenin, Myf5	MHC	Rotational rheometer
Xu et al.^40^	2019	Matrigel	PDMS mould	Human muscle precursor cells	7.5 ×10^6^ cells/ml	beta2 microglobulin, MyH1, MyH2, MyH3, MyH7, MyH8	PAX7, Alpha actinin	Force transducer
Nakayama et al.^41^	2019	Collagen	Not specified	C2C12, Human microvascular endothelial cells	5 ×10^5^ cells/scaffold	PAX7	MHC, Phalloidin, CD31	N/A
Bakooshli et al.^42^	2019	Geltrex	Nylon hooks	Human myoblasts, ESC-derived motor neuron clusters	1 ×10^5^ cells/cm^2^	N/A	MHC, Rapsyn, Alpha actinin, AChR	Force transducer
Yoshioka et al.^43^	2020	Collagen, matrigel	Polycarbonate cylinder	C2C12, murine iPS cells	3.2 ×10^5^ cells/well	N/A	Alpha actinin, Beta 3 tubulin	Muscle displacement by video microscopy
Xu et al.^44^	2020	Matrigel	Not specified	Human fibroblasts	1 ×10^7^ cells/ml	MyoG, DAG1, DMD, Actn2, Myh3, Myh7	MF20	Force transducer, Stimulated at 0.5, 1, 2, 5, 10, 20 and 40 Hz
Kim et al.^45^	2020	Collagen	Not specified	C2C12	1 ×10^7^ cells/ml	Myod1, Myh2, MyoD	DAPI, MHC, Alpha actinin	N/A
Kim et al.^46^	2020	Gelatin	PCL bioink	Human muscle progenitor cells, human neural stem cells	1 ×10^7^ cells/ml	N/A	MHC, Beta 3 tubulin, NF	N/A
Alave Reyes-Furrer et al.^47^	2021	Matrigel	Not specified	Primary human skeletal muscle-derived cells	2 ×10^7^ cells/ml	Myf5, MyoD, MyoG, Actn2, Myh1-3	MHC, Alpha actinin	Force transducer
Abdalkader et al.^48^	2021	Collagen	PDMS mould	C2C12	1 ×10^6^ cells/ml	N/A	MYH, DAPI, Phalloidin	Muscle displacement by microscopy
Bersini et al.^49^	2022	Gelatin	Steel rods	Human myoblasts, human fibroblasts	2.5 ×10^6^ cells/ml	PECAM-1, CD36	Alpha actinin, Dystrophin	N/A
Wells-Cembrano et al.^50^	2022	Matrigel	Velcro, PDMS	Human myoblasts	3 ×10^6^ cells/myobundle	N/A	SAA, MHC, AChR	Force transducer
Kim et al.^51^	2022	Collagen	Stainless steel pins	Human skeletal muscle, fibroblasts, endothelial cells	1 ×10^6^ cells/ml	N/A	Laminin	Force transducer
Pinton et al.^52^	2023	Matrigel	PDMS mould	Human iPSCs	2.5 ×10^3^ cells/cm^2^	MyoD, MHC, dys	MHC, Titin, PAX7, Laminin, CD31	Muscle displacement by microscopy
Shahriyari et al.^53^	2024	Matrigel	Stainless steel pins	Human iPSCs	3.2 ×10^6^ cells/ml	N/A	N/A	N/A
Shimizu et al.^54^	2017	Matrigel	PDMS pillar	C2C12	2 ×10^6^ cells/ml	Atrogin-1, Murph-1, beta actin	MHC	Muscle displacement by microscopy
Osaki et al.^55^	2018	Collagen, matrigel	PDMS pillar	Human iPSC-derived muscle cells, hESC neural cells	5 ×10^5^ cells/well	MyoD, Myh1, Myh2, Myl2	Alpha actinin, Beta 3 tubulin, GFAP, Occludin, ZO-1, Islet-1	Stage top incubator, axiovision
Davis et al.^56^	2019	Matrigel	PDMS mould	Human myoblasts, C2C12	7.5 ×10^5^ cells/myobundle	Myh1, Myh3, Myh7, Myh8, TATA	MHC, Hoechst, NF-kb	Force transducer
Nagashima et al.^57^	2020	Matrigel	PDMS mould	Human myogenic cells Hu5/KD3	Not specified	MyH1, MyH2, MyH7, MyH8	Alpha actinin	Force transducer
Fleming et al.^58^	2020	Matrigel	PDMS mould	Human muscle precursor cells	4 ×10^6^ cells/ml	PAX7, MYOD, MYOG	PAX7, MYOD1, Laminin	Force transducer
Rajabian et al.^59^	2021	Matrigel	Micropillars	Human myoblasts	3 ×10^6^ cells/myobundle	N/A	PAX7, Ki67, MHC, SAA	Muscle displacement by video microscopy
Shahriyari et al.^60^	2022	Matrigel	Not specified	Human pluripotent stem cells	1.3 ×10^4^ cells/cm^2^	PAX7, MYOD, MYOD1	PAX7, MYOD1, Myogenin	Force transducer

Table of included studies and model information.

### Histology and immunohistochemistry (IHC)

**Fig. 6:**
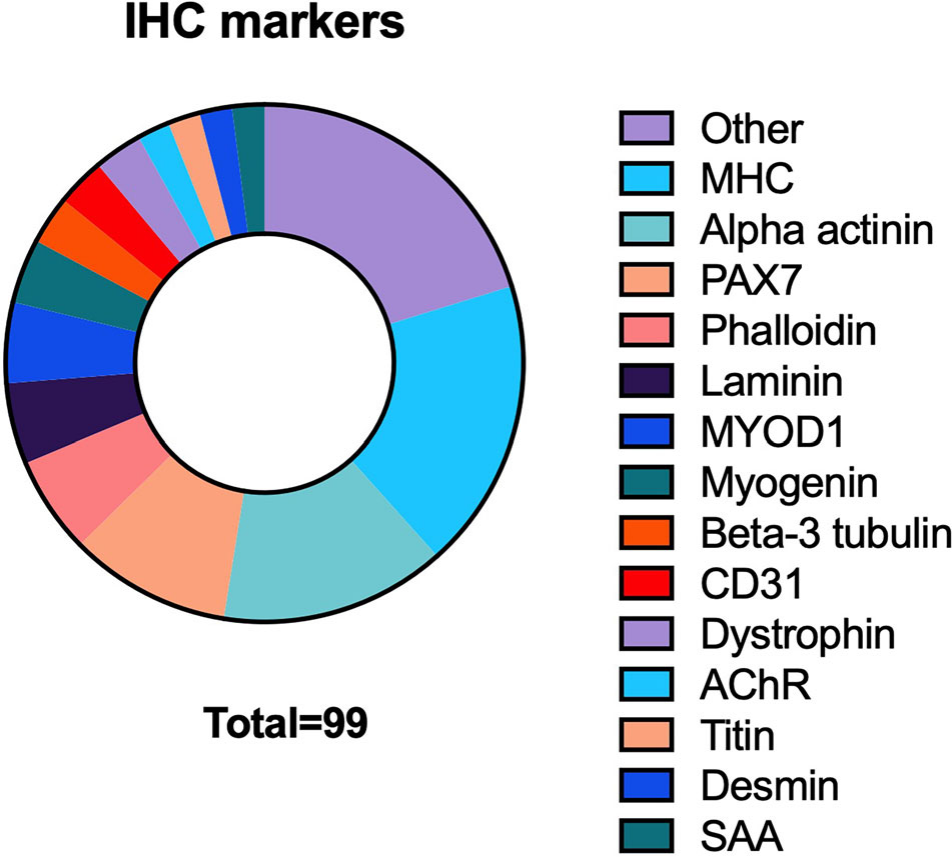
Immunohistochemistry (IHC) markers used for imaging. Where *n* = 1, markers have been grouped into the “other” category.

A range of different IHC stains used to characterise the models (Fig. 6). Myosin heavy chain (MHC), alpha actinin and Pax7 were the most common types of stains used for staining the muscle cells within the 3D constructs. Haematoxylin and eosin (H&E) was commonly used for general structural imaging.

### Quantitative polymerase chain reaction (qPCR)

**Fig. 7:**
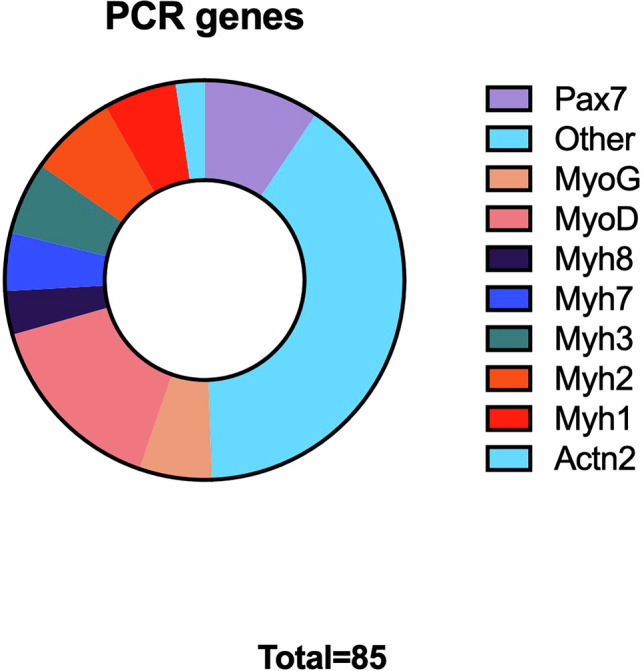
qPCR differentiation primers used amongst included studies. Where *n* = 1, primers have been grouped into the “other” category.

A wide variety (*n* = 85) of genes were expressed using qPCR to demonstrate model maturation. Myogenic differentiation 1, Myogenin and Paired box 7 genes were amongst the most common (Fig. 7).

### Force measurement

**Fig. 8:**
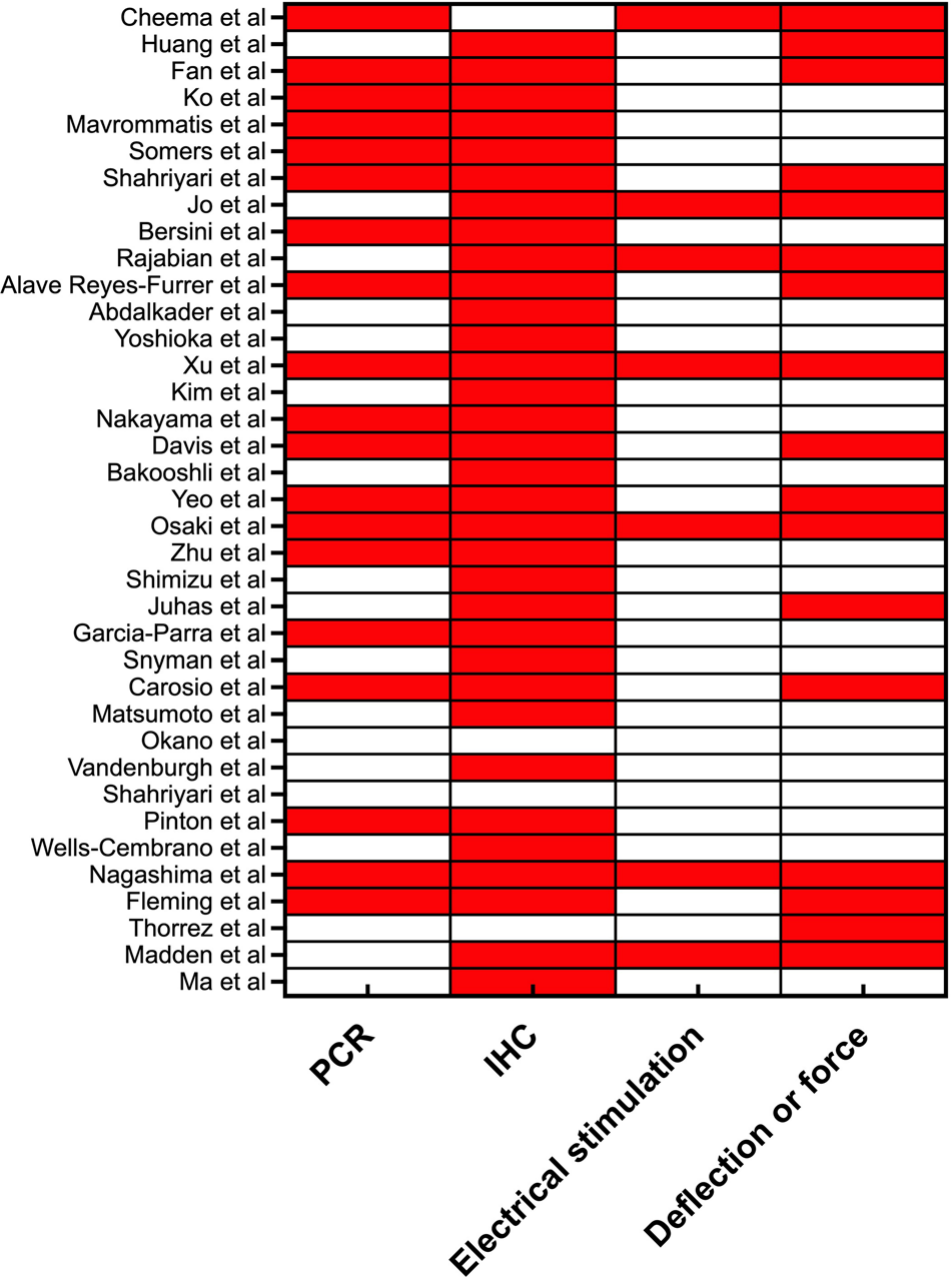
Outcome reporting in standard models. White – outcome measure not reported in experiment. Red – outcome measure reported in experiment.

Data for tetanus, twitch, specific force, and absolute force was reported across 3D skeletal muscle models (Table 2). Force was recorded either directly using a force transducer or indirectly by calculating the degree of displacement achieved by the model from a fixed cantilever post. Tetanus is defined as a sustained muscle contraction evoked when the model receives action potentials at a high rate. Muscle twitch is defined as a period of contraction and relaxation after a single stimulus. Absolute force is defined as the maximum force achieved by the model. Specific force is defined as force per unit of mass. The mean tetanus, twitch and absolute force reported was 3.72 mN, 4.51 mN and 0.23 mN, respectively. Studies were categorised according to the presence or absence of the aforementioned criteria (Fig. 8).

**Table 2:** Table of force generation

Study	Tetanic force	Units	Twitch	Units	Specific force	Units	Absolute force	Units
Cheema et al.							0.0509–0.1302	mN
Huang et al.	0.8059 ± 0.055	mN	0.329 ± 0.0263	mN				
Fan et al.					15.42 (Day 1) 147.67 (Day 7)	kPa		
Shahriyari et al.	2.8 ± 0.2	mN						
Reyes-Furrer et al.							0.4	mN
Xu et al.	0.748 ± 0.277	mN	0.374 ± 0.089	mN				
Juhas et al.	14.52 ± 1.55	mN	19.75 ± 2.01	mN				
Carosio et al.					0.893 ± 0.110	kPa		
Nagashima et al.	0.03	mN						
Fleming et al.	0.1016	mN	0.0366	mN	0.96	kPa		
Thorrez et al.					50-200	kPa		
Madden et al.	7.0 ± 2.2	mN	2.1 ± 0.9	mN				

Terminology is directly lifted from the published literature. The terminology of ‘specific force’ is used in literature and reported with units for pressure. Force has therefore been placed in quotation marks to highlight that the term force here has been used in this field to report pressure data. Absent data left blank.

Characteristics from standard models.

### Types of injury

**Fig. 9:**
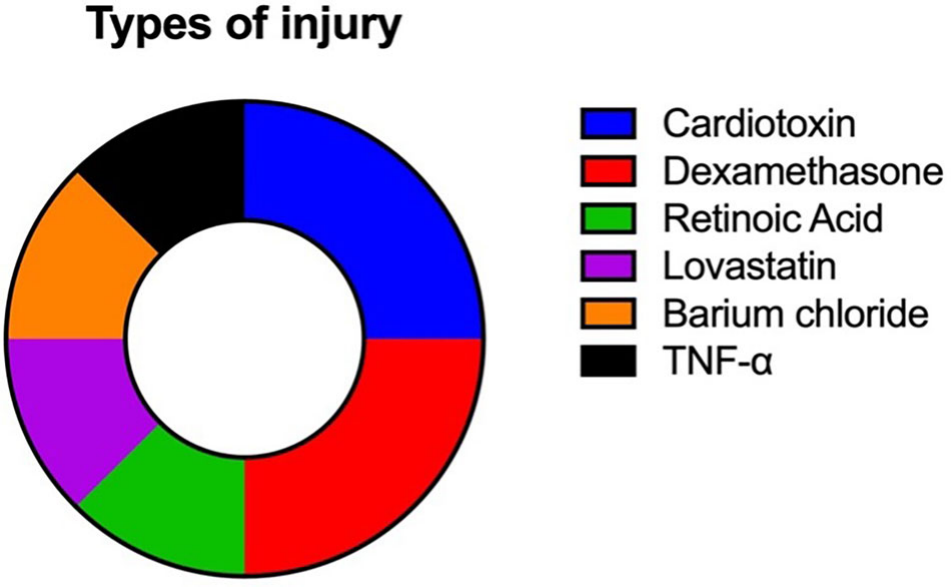
Types of injury which in vitro models were subjected to and relative frequency (*n* = 7). Doughnut chart depicting proportion of studies utilising specified injury.

7 of the included studies were subjected to injury. The most common injury mechanisms were using cardiotoxin (*n* = 2) and dexamethasone (*n* = 2) (Fig. 9).

### Bias assessment

5 included studies were deemed to have a moderate risk of bias using the OHAT tool. The remaining 31 studies had a low overall risk of bias (Table 3).

**Table 3:** Assessment of risk of bias of included studies using the OHAT tool

Authors	Year	Q1	Q2	Q3	Q4	Q5	Q6	Q7	Final outcome
Cheema et al.	2003	Low	Mod	Low	Low	Low	Low	Low	Low
Huang et al.	2004	Low	Mod	Low	Low	Low	Low	Low	Low
Bansai et al.	2019	Low	Mod	Low	Low	Low	Low	Low	Low
Fan et al.	2022	Low	Mod	Low	Low	Low	Low	Low	Low
Ko et al.	2023	Low	Mod	Low	Low	Low	Low	Low	Low
Mavrommatis et al.	2023	Low	Mod	Low	Low	Low	Low	Low	Low
Somers et al.	2022	Low	Mod	Low	Low	Low	Low	Low	Low
Shahriyari et al.	2022	Low	Mod	Low	Low	Mod	Low	Low	Mod
Jo et al.	2022	Low	Mod	Low	Low	Low	Low	Low	Low
Cakal et al.	2022	Low	Mod	Low	Low	Low	Low	Low	Low
Bersini et al.	2022	Low	Mod	Low	Low	Low	Low	Low	Low
Rajabian et al.	2021	Low	Mod	Mod	Low	Mod	Low	Low	Mod
Alave Reyes-Furrer et al.	2021	Low	Mod	Low	Low	Low	Low	Low	Low
Abdalkader et al.	2021	Low	Mod	Low	Low	Low	Low	Low	Low
Yoshioka et al.	2020	Low	Mod	Low	Low	Low	Low	Low	Low
Xu et al.	2020	Low	Mod	Low	Low	Low	Low	Low	Low
Kim et al.	2020	Low	Mod	Low	Low	Low	Low	Low	Low
Kim et al.	2020	Low	Mod	Low	Low	Low	Low	Low	Low
Nakayama et al.	2019	Low	Mod	Low	Low	Low	Low	Low	Low
Davis et al.	2019	Low	Mod	Mod	Low	Mod	Mod	Low	Mod
Bakooshli et al.	2019	Low	Mod	Low	Low	Low	Low	Low	Low
Yeo et al.	2018	Low	Mod	Low	Low	Low	Low	Low	Low
Osaki et al.	2018	Low	Mod	Low	Low	Low	Low	Low	Low
Zhu et al.	2017	Low	Mod	Low	Low	Low	Low	Low	Low
Shimizu et al.	2015	Low	Mod	Low	Low	Low	Low	Low	Low
Juhas et al.	2014	Low	Mod	Low	Low	Low	Low	Low	Low
Garcia-Parra et al.	2014	Low	Mod	Low	Low	Low	Low	Low	Low
Snyman et al.	2014	Low	Mod	Low	Low	Low	Low	Low	Low
Carosio et al.	2013	Low	Mod	Low	Low	Low	Low	Low	Low
Matsumoto et al.	2007	Low	Mod	Low	Low	Low	Low	Low	Low
Okano et al.	1997	Low	Mod	Low	Low	Low	Low	Low	Low
Vandenburgh et al.	1996	Low	Mod	Low	Low	Low	Low	Low	Low
Shahriyari et al.	2024	Low	Mod	Low	Low	Low	Low	Low	Low
Pinton et al.	2023	Low	Mod	Low	Low	Low	Low	Low	Low
Wells-Cembrano et al.	2022	Low	Mod	Low	Low	Low	Low	Low	Low
Kim et al.	2022	Low	Mod	Low	Low	Low	Low	Low	Low
Nagashima et al.	2020	Low	Mod	Low	Low	Low	Low	Low	Low
Fleming et al.	2020	Low	Mod	Mod	Low	Mod	Low	Low	Mod
Xu et al.	2019	Low	Mod	Low	Low	Low	Low	Low	Low
Thorrez et al.	2018	Low	Mod	Low	Low	Low	Low	Low	Low
Osaki et al.	2018	Low	Mod	Low	Low	Low	Low	Low	Low
Shimizu et al.	2017	Low	Mod	Mod	Low	Mod	Low	Low	Mod
Madden et al.	2015	Low	Mod	Low	Low	Low	Low	Low	Low
Ma et al.	2011	Low	Mod	Low	Low	Low	Low	Low	Low

### Modified Delphi study

The first questionnaire regarding the generation of a COS was sent out to 32 eligible corresponding authors. The initial response rate was 25% (*n* = 8). A further refinement questionnaire was sent one month later with a response rate of 50% (*n* = 4). A final COS was generated based on all responses (Table 4).

**Table 4:** Proposed core outcome table for any three-dimensional in vitro skeletal muscle model

Criteria	Morphology and phenotype	Function
Essential	•Timeline showing evolution of model morphology (*pictorial over time)* •Myotube length and diameter •Immunohistochemical antibodies to sarcomeric protein, namely myosin heavy chain (MHC), actin, tropomyosin •Confirmation of striation through microscopy •Maximum demonstrated lifespan of muscle model (total time in culture) •Percentage of parallel alignment of muscle fibres measures in percentage per field	•Description of anchoring method and materials • •Axial strain applied •Specific contractile force (tetany) •Specific twitch force •Applied voltage •Pulse width •Distance between electrodes
Desirable	•Cell death-related indicators – percentage nuclei loss per myotube and changes in myotube length and diameter •Neural or endothelial staining if multiple cell types are used in the model •Cell populations within model and quantity at full maturation •Cell seeding densities at start of model development •Quantification of collagen lay down through IHC	•Glucose metabolism measured using radiolabelled glucose •Calcium flux using fluorometry

It is envisaged that having the above “baseline” will allow researchers to compare models with greater accuracy. The table is not exhaustive, rather suggestive of a uniform starting point. All quantifiable data should be reported in SI units.

## Discussion

**Fig. 10:**
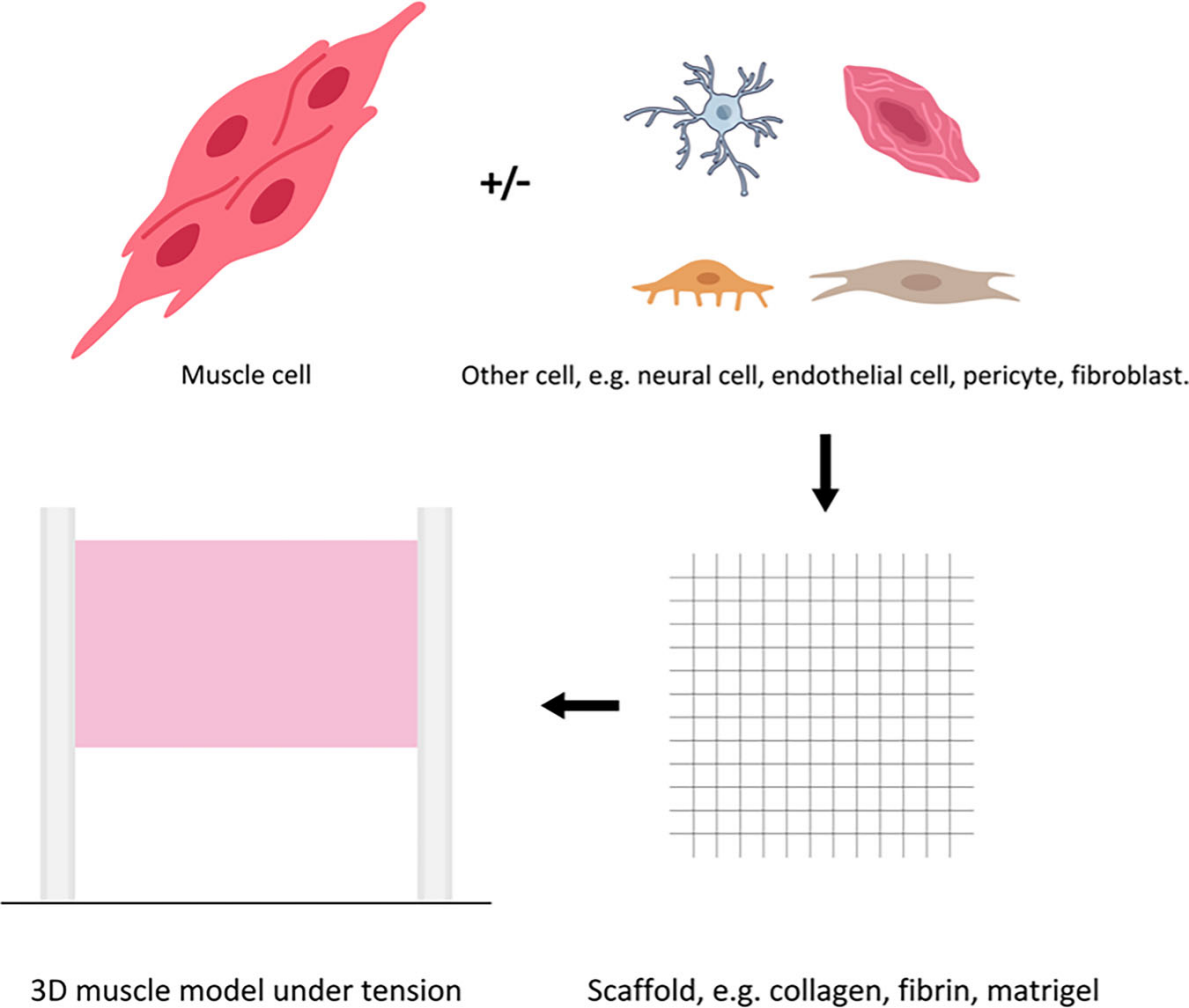
Schematic highlighting the basic constituents of an in vitro 3D muscle model. Multicellular models included a variety of cells, with some incorporating neuromuscular junctions and vascular networks.

This systematic review evaluated current literature on in vitro 3D skeletal muscle models under tension and reveals substantial variability in how these systems are constructed (Fig. 10), validated, and reported. Although all models demonstrated expression of muscle-specific genes and proteins, and some achieved measurable force generation, there remains considerable inconsistency in cell composition, spatial organisation and methodological standardisation. These discrepancies limit comparability and impede translation of findings. Drawing from these observations, we propose a COS to unify reporting practices and facilitate benchmarking across future studies.

The diversity of cell lines employed to generate 3D muscle constructs underscores the challenge of reproducing the complex architecture of native human skeletal muscle. Most studies relied heavily on murine C2C12 myoblasts due to their accessibility and scalability, while human muscle progenitor cells (MPCs) and primary rat cells were less frequently used. However, achieving structural and functional fidelity requires the integration of multiple cell types that collectively establish the multicellular muscle niche. Endothelial cells, fibro-adipogenic progenitors (FAPs), immune cells, and pericytes each play distinct roles in vascularisation, extracellular matrix (ECM) remodelling, and regeneration^8^.

A key determinant of construct fidelity is the ratio and spatial arrangement of myogenic and supporting cell populations. These proportions influence differentiation trajectories and the hierarchical organisation of the developing tissue. Despite their importance, quantitative optimisation of cell ratios is rarely reported. For example, endothelial cell inclusion has been shown to enhance myogenesis and repair via microvascular mimicry^9^, yet no consensus exists regarding effective seeding densities. The absence of standardised compositional frameworks likely contributes to the variability observed across model morphology and function.

Pluripotent stem cell (PSC)–derived models have begun to address these limitations by capturing self-organising developmental dynamics. Induced pluripotent stem cell (iPSC)-based systems, in particular, generate subpopulations with proliferative and differentiative potential, offering closer alignment with in vivo myogenesis^10^. Nevertheless, replicating the full cellular complexity of native muscle—including pericytes, FAPs, and immune elements—remains an ongoing challenge.

Spatial organisation is equally critical to functional maturation. Engineered microtopographies that promote cellular alignment have been shown to enhance myotube structure and contractile output^11^. Yet, detailed assessment of alignment and microarchitecture is inconsistently reported, despite their established relevance to tissue integrity and biomechanics^12^. Collectively, these findings highlight that future progress in muscle tissue engineering will depend on integrating cellular diversity with controlled spatial patterning and quantitative evaluation of structure–function relationships.

Immortalised cell lines, particularly the murine C2C12 myoblast, continue to dominate the field due to their accessibility, rapid proliferation, and tolerance to extended culture. Their genetic stability and scalability make them well suited for high-volume experiments and reproducibility. However, these advantages are counterbalanced by fundamental biological limitations. The process of immortalisation disrupts normal cell cycle regulation and alters gene expression, producing metabolic and phenotypic deviations from primary myogenic cells^13^. Consequently, immortalised lines may represent a distinct, non-physiological cell type.

Co-culturing immortalised cells with other lineages has been shown to partially restore structural and functional features. Constructs combining C2C12 cells with non-myogenic populations displayed improved fibre alignment, myotube maturation, and force generation, reinforcing the importance of cellular cross-talk. Nevertheless, few studies have adopted alternative immortalised human lines, such as AB1167, despite their stable phenotype and potential as standardised control systems^14–16^.

Immortalised models also lack a functional satellite cell niche—a hallmark of regenerative capacity. In contrast, MPCs and iPSC-derived myogenic cells exhibit more advanced differentiation, characterised by higher myosin heavy chain (MHC) expression, defined cross-striations, and enhanced responsiveness to electrical stimulation^17^. These features more closely mirror the structure and function of native muscle, suggesting that primary or pluripotent-derived populations are better suited for disease modelling and regenerative studies. Recent advances incorporating multiple stem cell–derived lineages have produced constructs with refined architecture, aligned myofibres, and quantifiable force output^17^. The growing adoption of human iPSCs since 2023 indicates a clear shift toward renewable and physiologically relevant cell sources, potentially establishing pluripotent systems as the new baseline for scalable muscle bioengineering.

Tension is a fundamental requirement for in vitro skeletal muscle maturation. Most reviewed models employed polydimethylsiloxane (PDMS) anchors due to their biocompatibility, accessibility, and ease of fabrication. Although effective for establishing tensioned constructs, PDMS only approximates the bone–tendon–muscle continuum found in vivo. More physiologically representative approaches—such as mineralised or bone-mimetic anchors—may improve structural fidelity^18,19^.

In vivo, mechanical tension arises through dynamic interactions among bones, tendons, and contractile fibres. Replicating this in vitro demands stable, load-bearing supports and ECM analogues capable of transmitting force. Collagen remains the most widely used ECM substitute due to its load-bearing and bioactive properties^20^. However, its pH sensitivity during neutralisation and variability in isoform composition introduce inconsistencies in gel stiffness and reproducibility. These differences can profoundly influence satellite cell recruitment and regenerative outcomes.

Synthetic materials such as alginate, silicone, and polyethylene glycol (PEG) offer tuneable mechanical characteristics and reproducibility but lack biological relevance. While these polymers show promise for synthetic muscle fabrication and volumetric loss repair, their utility for modelling physiological muscle remains limited. Future work should integrate hybrid materials that combine mechanical precision with biochemical functionality, supporting both tensile load and cellular signalling.

Functional validation through force measurement remains a cornerstone of model assessment. Most studies employed cantilever post deflection assays, inferring force via Euler beam theory. However, methodological inconsistencies were common. Several studies incorrectly assumed uniform load distribution despite evidence that hydrogels apply concentrated or partially distributed forces at the post tip, potentially leading to overestimated deflection values. Although the magnitude of this error may be modest, it introduces systematic uncertainty and underscores the need for standardised analytical frameworks.

Notably, no studies measured deflection or force output following injury, an omission that limits evaluation of regenerative capacity. Monitoring mechanical recovery after insult could serve as a functional correlate of repair, complementing electrophysiological metrics such as twitch and tetanic contraction. In addition, voltage parameters used to elicit contractions varied widely, complicating cross-study comparisons. Harmonising electrical stimulation protocols—including voltage amplitude, pulse duration, and frequency—would substantially improve reproducibility.

Reporting of contractile data also lacks uniformity. Some groups report absolute force, others specific force normalised to cross-sectional area, but few provide both. In physiological muscle research, specific tension is the standard metric of functional maturity. Adopting similar conventions in engineered systems would enable direct comparison with native tissue benchmarks. Standardised reporting of active and passive force parameters is therefore essential to assess true biomechanical equivalence.

**Fig. 11:**
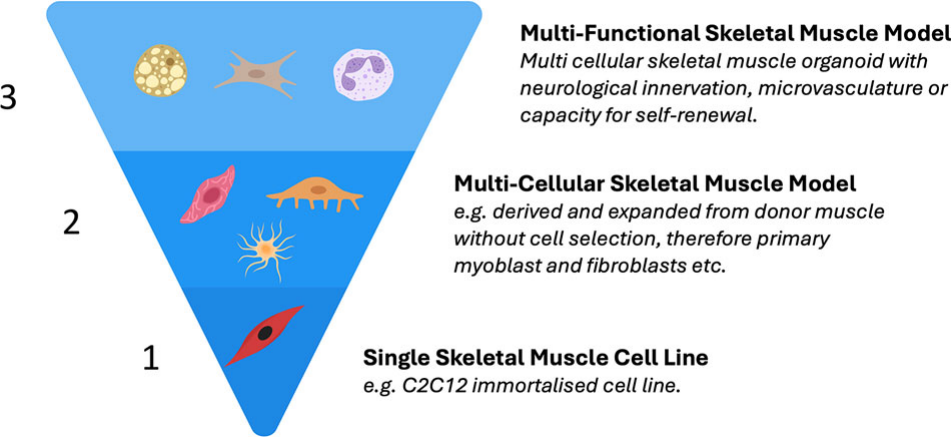
Hierarchy of heterogeneity and complexity of model. Models that have only muscle cells are most primitive and at level 1 of the index, level 2 includes at least one other cell line and level 3 includes 2 or more with an advanced capability such as the ability to self-renew.

The generation of multicellular muscle constructs that recapitulate native architecture and functionality remains a central ambition of tissue engineering. Whether derived from MPCs or iPSCs, these systems seek to emulate the complex interplay among contractile, vascular, stromal, and immune components that sustain muscle homeostasis in vivo. However, this biological hierarchy is difficult to reproduce experimentally, as it requires simultaneous coordination of mechanical and biochemical cues (Fig. 11).

**Fig. 12:**
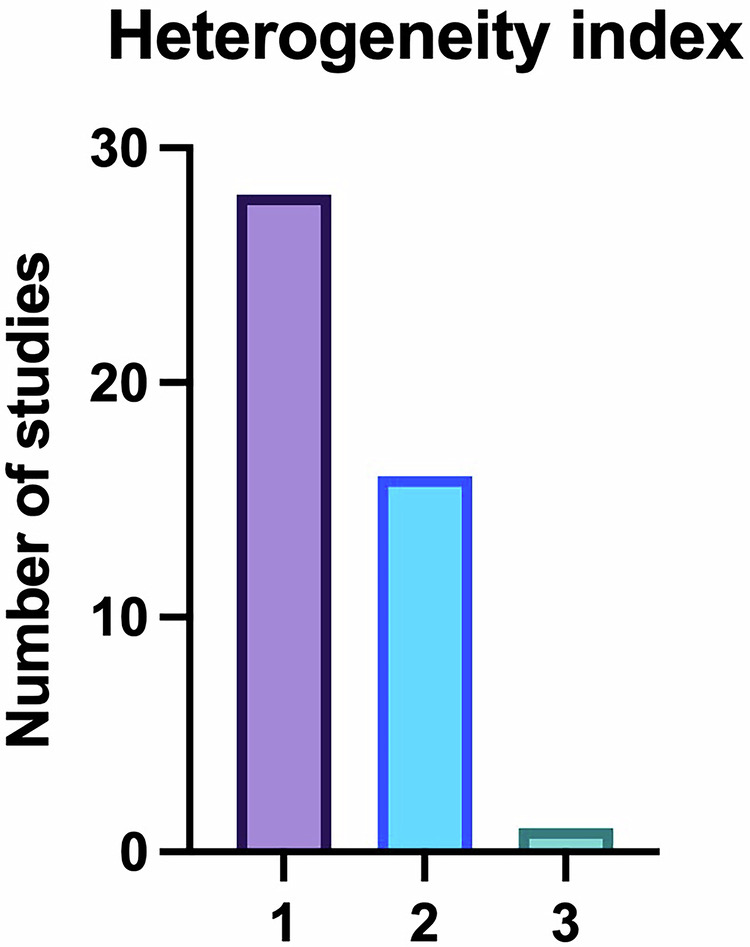
Number of studies by heterogeneity index. Bar depicts number of studies with specified heterogeneity index.

High-fidelity multicellular systems represent one end of a continuum of model sophistication. While such models are indispensable for studying disease mechanisms and regeneration, simpler constructs continue to provide critical insights into mechanistic pathways and serve as efficient screening platforms. Recognising this hierarchy of heterogeneity allows researchers to align experimental goals with model complexity and interpret outcomes appropriately (Fig. 12). Establishing this conceptual framework clarifies which structural and molecular parameters should be reported universally, and which apply only to advanced models, enabling coherent benchmarking across the field.

A persistent issue identified in this review is the absence of standardised criteria for model validation and outcome reporting. The methods used to define and verify muscle differentiation vary widely, impeding cross-comparison. Most studies used qualitative staining (e.g., H&E, phalloidin, or MHC) and quantitative gene expression of Pax7, MyoD, and myogenin. However, these markers are temporally regulated and context dependent. A standardised panel of terminal differentiation markers—including MHC isoforms, sarcomeric α-actinin, and dystrophin—would provide a more reliable measure of maturity and allow comparison between studies.

Before force quantification, morphological metrics such as fusion index, myotube alignment, and fibre-type composition should be verified to ensure functional tests reflect mature tissue behaviour. Integrating transcriptomic or proteomic profiling could further validate maturity, as discrepancies between mRNA and protein levels are common in vitro. Proteomic signatures may, in fact, represent a more robust indicator of terminal differentiation, and comparative profiling against in vivo muscle tissue could provide physiologically meaningful benchmarks^19^.

Standardisation is also lacking in models of injury and regeneration. In vivo, skeletal muscle repair is mediated by satellite cell activation, macrophage recruitment, and cytokine release (e.g., IL-6, TNF-α, TGF-β)^21^. In vitro, most studies employed chemical insults such as cardiotoxin, barium chloride, or TNF-α to induce damage^22^. These approaches reliably disrupt cell integrity but fail to replicate the inflammatory and necrotic cascades of physiological trauma. Mechanical or crush-based injury—well established in 2D culture and animal models—has yet to be systematically explored in 3D systems^23^.

Future frameworks should characterise injury in three key domains: the dose–response relationship of the applied insult; the mechanism of cell death (necrosis, apoptosis, or necroptosis); and the regenerative capacity of the construct post-injury (Table 5). Quantitative assays such as lactate dehydrogenase (LDH) release, myogenic regulatory factor re-expression, and the proportion of necrotic cells could form a minimal dataset for evaluating repair. Without such consistency, comparative interpretation across studies remains speculative.

**Table 5:** Considerations for data presentation of injury effects on 3D skeletal muscle models

Domain	Injury	Outcome quantification
Dose and effect	How is the dose and injury response titrated for the model?	What is the degree of damage being inflicted on the model – LD50? *Changes in cell metabolism?* *LDH release?*
Mechanism	By what mechanism is the stimulus causing cell death or disruption? Can this be quantified? By what mechanism are the cells dying e.g. necrosis, necroptosis, apoptosis?	How does this stimulus effect the cell? Changes in ECM? Structural morphology? RNA/protein changes? Overall function?
Regeneration	How does the model respond to injury?	Through what cells does the model regenerate? What structural changes are seen? ECM/collagen? *Changes in myotube morphology?* *Altered collagen subtypes?* *Satellite cell population changes?*

To address these challenges, we propose a COS for 3D skeletal muscle models under tension (Table 4). This framework defines essential structural, molecular, and functional benchmarks—including MHC, Pax7, and α-actinin expression, alongside specific twitch and tetanic force measurement. Incorporation of myogenic regulatory factors (MRFs) through qPCR or RNA sequencing further ensures lineage fidelity. Establishing a unified baseline for outcome reporting will promote reproducibility, enable direct model comparison, and accelerate the refinement of systems that more accurately reproduce human muscle physiology.

### Proposed core outcome set for 3D skeletal muscle models

There are limitations to the methodology. Much of the in vitro literature is reported in the Web of Science database, rather than on MEDLINE or EMBASE. The former database does not use the keyword structure as MEDLINE or EMBASE databases. Therefore, it is necessary to translate a search strategy between databases. This could introduce some errors and inconsistencies into the methodology. The approach in this review has thus attempted to reduce errors and achieve a reproducible strategy across all databases. Although this work adopted the OHAT risk of bias tool, more work is required to develop a robust technique to assess and report bias amongst in vitro literature. This is pertinent for questions 5 and 6, which focus on outcome assessment and reporting. The use of randomisation is rare in in vitro studies, and adopting this question within the OHAT perhaps scores unnecessary bias. Focus on cell types, seeding numbers, replicates and power calculations may be more useful ways of assessing bias as well as reproducibility within the in vitro literature.

This systematic review outlines the current research in the field of 3D skeletal muscle models under tension. It demonstrates the variety of cell types used, the techniques used to construct models and the functional and morphological outcomes reported. We highlight the fact that the field should be aiming for increasingly complex models that are sufficiently heterogenous and reflect in vivo muscle physiology. Finally, through a modified Delphi process, we propose a novel COS for the reporting of in vitro 3D muscle models under tension. By agreeing on a uniform standard of reporting, we envisage ease of comparison between models whilst simultaneously allowing the researcher freedom with regards to “how” the model is engineered. Future researchers will be able to validate their own models using this COS prior to subjecting them to different interventions. The ultimate aim of this study is to collate high-quality empirical evidence and make it translatable for understanding disease mechanisms and facilitating drug discovery.

## Methods

This systematic review was reported in compliance with the PRISMA 2020 checklist. The protocol was developed prospectively in collaboration with a research librarian (NT) at the Nuffield Department of Rheumatology and Musculoskeletal Sciences (NDORMS). The following search protocol was published on www.protocols.io.

All databases were searched from inception until 13/03/2024. The Ovid interface was used to search MEDLINE (1946-present), EMBASE (1974-present) and Web of Science Core Collection (1900-present) databases. No date or language limits were applied. Synonyms were produced in line with elements of the research question. “Skeletal muscle” was the major inclusion criterion, followed by “three dimensional” and “model”. Full search strategies can be found in the appendix.

### Inclusion criteria

3D in vitro studies that utilised any skeletal muscle cell lines (primary or immortalised, single or multicellular) of any species cultured with an anchor system imposing axial strain in order to mimic a native muscle-bone interface were eligible for inclusion. Models that used organic hydrogel compounds native to the human body (fibrin or collagen) were included. Where studies reported minor experimental modifications of previously reported models (with detailed methods), the most comprehensive study or studies were included. Self-assembly models were also included. Included studies also had to demonstrate force production, which is a critical functional element of skeletal muscle.

### Exclusion criteria

Models that did not impose axial strain, self-assembly models (spheroids) or tissue explant models (including slice culture) without axial strain, were excluded. There was no limitation on the magnitude of applied tension to the model. In vitro monolayer systems were excluded. Models incorporating hydrogels using materials that were incapable of biodegradation (breakdown of organic matter into constituent elements) within human tissue were excluded. Studies without detailed methods, opinion pieces, reviews, letters, conference abstracts, in vivo work, and book chapters were excluded. Studies describing previously published protocols were also excluded.

### Study selection and screening process

References were initially exported to Endnote 21 for de-duplication (Endnote X9, Clarivate Analytics, USA). Search results were de-duplicated and exported directly to Rayyan (www.rayyan.ai) for screening. Initial abstract screening was undertaken by two authors (KB and RS). Abstracts were first screened blindly on Rayyan and where there was conflict, a discussion regarding article suitability took place. Secondary screening was undertaken by examining the full texts of all included abstracts. The bibliographies of included articles were also screened for further relevant studies which detailed original 3D in vitro muscle models (Fig. 1).

### Outcome measures

The primary outcome measure was the type of model used. Data collected included type of material, nature of the anchor system, material(s) concentration, crosslinking material and concentration, and cell seeding concentration. Secondary outcome measures were techniques utilised to define the muscle model. Muscle models were then inspected for whether they were injured and tertiary outcome measure was the type of injury induced.

### Quality assessment

The risk of bias was assessed by a single author (KB) and reviewed with the senior author (RS) using the office of health assessment and translation (OHAT) risk of bias rating tool for human and animal studies^6^. This tool has been amended to allow assessment of in vitro studies. The tool evaluates the evidence along seven bias questions (Table 6). Each question was given a low, moderate or high bias rating. Overall bias was then calculated based on the proportion of bias found in the majority of questions.

**Table 6:** The OHAT risk of bias questions^5^

Number	Question
Q1	Same experimental conditions
Q2	Blinding during study
Q3	Incomplete data
Q4	Exposure characterisation
Q5	Outcome assessed
Q6	Outcome reporting
Q7	Other

Each question evaluated whether the study applied these experimental approaches.

### Modified Delphi method for COS generation

**Fig. 13:**
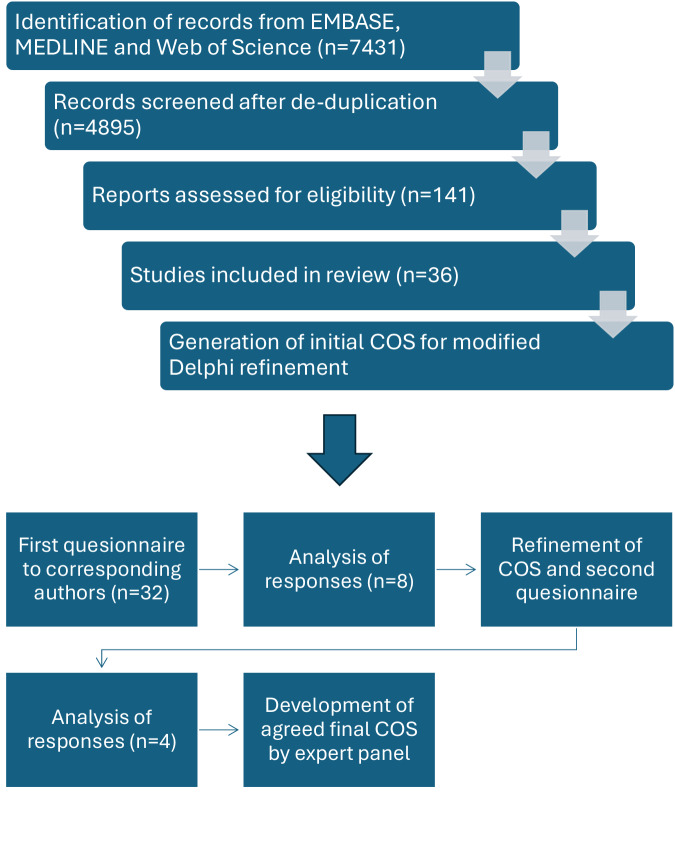
A modified Delphi process for the selection of a COS group and its refinement process. Flowchart depicts iterative process leading to the final COS.

Novel methodology for the synthesis of in vitro 3D skeletal muscle models was used for this study (Fig. 13). The Delphi method was originally developed to obtain a reliable consensus amongst a group of experts^7^. We present a modified approach as the selection of experts has been through the inclusion of the corresponding authors of included studies. This theoretically reduces bias, as the initial selection of experts for the Delphi process was not opinion based.

## Supplementary information

Supplemental information

Appendix

## Data Availability

All data and results associated with the dataset are available in the main text and in the Supplementary Information. Any other material associated with this paper will be shared upon reasonable request.
