# Supplementary material for: Three-dimensional models of skeletal muscle under tension: a systematic review and core outcome set
Source: NPJ Regen Med. 2026 Apr 14;11:29. doi: 10.1038/s41536-026-00464-z (PMC13269917; doi:10.1038/s41536-026-00464-z)
Supplement: Supplementary file 2 — Appendix [file 41536_2026_464_MOESM2_ESM.pdf]

Appendix:

Total Records downloaded (enter this number here to update SearchReport tab): 197Search Strategy

Embase 1974 to present

1	skeletal myoblast/	596
2	(skeletal adj2 myoblast*).tw,kf.	2170
3	skeletal muscle cell line/	83
4	(cell* adj2 (stem or primary or satellite)).tw,kf.	624478
5	(myogenic or skeletal muscle).tw,kf.	172977
6	4 and 5	11204
7	skeletal muscle/	144693
8	slow muscle fiber/	2149
9	fast muscle fiber/	2212
10	skeletal.tw,kf.	299053
11	(muscle* adj2 fiber*).tw,kf.	35259
12	10 and 11	13999
13	(fast-twitch or intermediate or white or type II).tw,kf.	1117798
14	(red or slow-twitch or type I).tw,kf.	630013
15	13 or 14	1657758
16	11 and 15	6707
17	myotube*.tw,kf.	15390
18	(skeletal adj2 myocyte*).tw,kf.	751
19	(myogenic or skeletal muscle).tw,kf.	172977
20	(cell* adj2 c2c12).tw,kf.	6130
21	19 and 20	3398
22	1 or 2 or 3 or 6 or 7 or 8 or 9 or 12 or 16 or 17 or 18 or 21	171236
23	3d.tw,kf.	324905
24	three d.tw,kf.	638

25	3 dimension*.tw,kf.	38685
26	organoid/	13217
27	organoid*.tw,kf.	23541
28	exp lab on a chip/	11240
29	lab on a chip.tw,kf.	3765
30	organ on a chip.tw,kf.	1453
31	exp bioengineering/	235895
32	bioengineer*.tw,kf.	15596
33	biological engineer*.tw,kf.	1427
34	tissue engineer*.tw,kf.	80668
35	23 or 24 or 25 or 26 or 27 or 28 or 29 or 30 or 31 or 32 or 33 or 34	628442
36	22 and 35	3934
37	model*.tw,kf.	5000672
38	scaffold*.tw,kf.	158968
39	architecture*.tw,kf.	197564
40	platform*.tw,kf.	357571
41	(system or systems).tw,kf.	4417710
42	37 or 38 or 39 or 40 or 41	8919326
43	36 and 42	2505
44	conference*.pt.	5859448
45	43 not 44	1970

Total Records downloaded (enter this number here to update SearchReport tab): 2650

Search Strategy

Medline (Ovid MEDLINE® Epub Ahead of Print, In-Process & Other Non-Indexed Citations, Ovid MEDLINE® Daily and Ovid MEDLINE®) 1946 to present

1	exp Myoblasts, Skeletal/	4090
2	(skeletal adj2 myoblast*).tw,kf.	1795
3	(cell* adj2 (stem or primary or satellite)).tw,kf.	422521
4	(myogenic or skeletal muscle).tw,kf.	139662
5	3 and 4	8576
6	Muscle, Skeletal/	166516
7	Muscle Fibers, Skeletal/	17604
8	Muscle Fibers, Slow-Twitch/	2488
9	Muscle Fibers, Fast-Twitch/	3131
10	skeletal.tw,kf.	237495
11	(muscle* adj2 fiber*).tw,kf.	29880
12	10 and 11	11474
13	(fast-twitch or intermediate or white or type II).tw,kf.	844075
14	(red or slow-twitch or type I).tw,kf.	493609
15	13 or 14	1266664
16	11 and 15	5555
17	myotube*.tw,kf.	12495
18	(skeletal adj2 myocyte*).tw,kf.	600
19	(myogenic or skeletal muscle).tw,kf.	139662
20	(cell* adj2 c2c12).tw,kf.	4658
21	19 and 20	2556
22	1 or 2 or 5 or 6 or 7 or 8 or 9 or 12 or 16 or 17 or 18 or 21	193945
23	3d.tw,kf.	249537

24	three d.tw,kf.	468
25	3 dimension*.tw,kf.	29970
26	Organoids/	13773
27	organoid*.tw,kf.	15510
28	Lab-On-A-Chip Devices/	7533
29	lab on a chip.tw,kf.	3690
30	organ on a chip.tw,kf.	1382
31	exp Bioengineering/	62819
32	bioengineer*.tw,kf.	12040
33	biological engineer*.tw,kf.	432
34	Tissue Engineering/	45681
35	tissue engineer*.tw,kf.	64407
36	23 or 24 or 25 or 26 or 27 or 28 or 29 or 30 or 31 or 32 or 33 or 34 or 35	387881
37	22 and 36	3428
38	model*.tw,kf.	3967863
39	scaffold*.tw,kf.	135360
40	methods.fs.	4360174
41	architecture*.tw,kf.	172933
42	platform*.tw,kf.	271052
43	(system or systems).tw,kf.	3641694
44	38 or 39 or 40 or 41 or 42 or 43	10499757
45	37 and 44	2650

Total Records downloaded (enter this number here to update SearchReport tab): 2811

Search Strategy

Search Query Database Results Date Run

1 "myotube OR (skeletal NEAR/2 myoblast*) OR ""skeletal muscle"" OR ""fast twitch muscle fiber*"" OR ""slow twitch muscle fiber*"" OR (skeletal NEAR/2 myocyte*) (Topic) AND 3d OR ""three dimension*"" OR ""three d"" OR organoid* OR ""lab on a chip"" OR ""organ on a chip"" OR bioengineer* OR ""tissue engineer*"" OR ""biological engineer*"" (Topic) AND model* OR scaffold* OR architecture* OR platform* OR system OR systems (Topic) " Web of Science Core Collection 2811 Wed Mar 13 2024 16:07:41 GMT+0000 (Greenwich Mean Time)

Full list of databases for Web of Science (to be included in appendices)- WOS.IC: 1993 to 2024 - WOS.CCR: 1985 to 2024 - WOS.SCI: 1900 to 2024 - WOS.AHCI: 1975 to 2024 - WOS.BHCI: 2005 to 2024 - WOS.BSCI: 2005 to 2024 - WOS.ESCI: 2015 to 2024 - WOS.ISTP: 1990 to 2024 - WOS.SSCI: 1900 to 2024 - WOS.ISSHP: 1990 to 2024
